# ODTbrain: a Python library for full-view, dense diffraction tomography

**DOI:** 10.1186/s12859-015-0764-0

**Published:** 2015-11-04

**Authors:** Paul Müller, Mirjam Schürmann, Jochen Guck

**Affiliations:** Biotechnology Center of the TU Dresden, Tatzberg 47-51, Dresden, 01307 Germany

**Keywords:** Refractive index, Single-cell analysis, Diffraction tomography, Backprojection, Backpropagation, Rytov, Born, Radon

## Abstract

**Background:**

Analyzing the three-dimensional (3D) refractive index distribution of a single cell makes it possible to describe and characterize its inner structure in a marker-free manner. A dense, full-view tomographic data set is a set of images of a cell acquired for multiple rotational positions, densely distributed from 0 to 360 degrees. The reconstruction is commonly realized by projection tomography, which is based on the inversion of the Radon transform. The reconstruction quality of projection tomography is greatly improved when first order scattering, which becomes relevant when the imaging wavelength is comparable to the characteristic object size, is taken into account. This advanced reconstruction technique is called diffraction tomography. While many implementations of projection tomography are available today, there is no publicly available implementation of diffraction tomography so far.

**Results:**

We present a Python library that implements the backpropagation algorithm for diffraction tomography in 3D. By establishing benchmarks based on finite-difference time-domain (FDTD) simulations, we showcase the superiority of the backpropagation algorithm over the backprojection algorithm. Furthermore, we discuss how measurment parameters influence the reconstructed refractive index distribution and we also give insights into the applicability of diffraction tomography to biological cells.

**Conclusion:**

The present software library contains a robust implementation of the backpropagation algorithm. The algorithm is ideally suited for the application to biological cells. Furthermore, the implementation is a drop-in replacement for the classical backprojection algorithm and is made available to the large user community of the Python programming language.

**Electronic supplementary material:**

The online version of this article (doi:10.1186/s12859-015-0764-0) contains supplementary material, which is available to authorized users.

## Background

The measurement of the refractive index of a biological cell is always connected to the observable phase change of light as it passes through the cell. For example, phase contrast microscopy in combination with refractive index matching is used to obtain an average value for the refractive index of a cell population [1]. Accuracy and flexibility of this approach are greatly improved with quantitative phase imaging techniques, such as digital holographic microscopy (DHM) [2]. Nevertheless, the reconstruction of the refractive index with sub-cellular resolution and in three dimensions requires a tomographic approach.

### Optical projection tomography

Optical projection tomography (OPT) is a well-studied technique that is used to reconstruct and quantify volumetric data of biological specimens [3, 4]. What distinguishes OPT from conventional 3D imaging techniques in biology, such as e.g. confocal microscopy or selective plane illumination microscopy (SPIM), is the type of measurement data that is acquired and the way it is processed to reconstruct the 3D measurement volume. Tomographic data sets (sinograms) consist of multiple angular *projections* of the 3D specimen, whereas the techniques mentioned above directly acquire *sclices* of the reconstruction volume. In OPT-based refractive index measurements, the projections from multiple angles are quantitative phase images. From these phase projections, the 3D refractive index can be reconstructed by means of the filtered backprojection algorithm [5, 6], an efficient algorithm that computes the inverse Radon transform. In the context of this paper, we refer to the filtered backprojection algorithm as the Radon approximation, because it assumes that light travels along straight lines. The Radon approximation is only valid for small wavelengths (e.g. x-ray radiation). Thus, when light with wavelengths in the visible spectrum (e.g. 400–600 nm) propagates through a biological cell, with structures on similar length scales, diffraction takes place and significant interference effects emerge.

### Optical diffraction tomography

Optical diffraction tomography (ODT) takes this wave nature of light into account. The diffraction of waves at objects is described by the Helmholtz equation. To simplify the complex description of waves by the Helmholtz equation, two known approximations are commonly applied; the Born and Rytov approximations. While the Born approximation assumes that only a small fraction of the wave is diffracted by the cell, the Rytov approximation assumes that the local refractive index variation inside the cell is small. The Rytov approximation is known to be superior to the Born approximation in ODT [7], but its implementation requires a phase unwrapping step that is not present in the implementation of the Born approximation. Note that OPT with the Radon approximation requires a similar phase unwrapping step. Furthermore, OPT operates on the measured phase data only, whereas ODT additionally incorporates the amplitude data in the reconstruction process. The principles of ODT were first introduced in [8] and a backpropagation algorithm was then described in [9]. The theoretical basis of ODTbrain, as well as a derivation of the full 3D backpropagation algorithm in the Rytov approximation are given in [6].

### Our contribution

We present what to our knowledge is the first publicly available software implementation of the 3D backpropagation algorithm. The algorithm can be used to reconstruct 3D refractive index maps from projections of biological or artificial phase objects. We showcase our implementation by reconstructing simulated cells and comparing the reconstruction qualities of OPT (Radon) and ODT (Born, Rytov). Furthermore, we investigate the contribution of several physical parameters to the overall reconstruction quality, complement previously conducted two-dimensional (2D) studies [7, 10, 11] with 3D data, and draw conclusions concerning the validity of the backpropagation algorithm in three dimensions.

## Implementation

The ODTbrain library is implemented in the Python programming language. To make the reconstruction process more transparent and to allow user-defined modifications of the reconstruction, we split the reconstruction process into three steps: a) apply a filter to the complex wave sinogram that corresponds to the required approximation (Born, Radon, or Rytov), b) reconstruct the object data from the sinogram (backproject or backpropagate), c) compute the refractive index distribution from the obtained object data. A full documentation, including a method reference and multiple examples, is available at the project home page.

The filtering step (a) is necessary for the application of the Radon and Rytov approximations. For 3D data sets, we rely on the 2D phase-unwrapping algorithm described in [12]. Step (b) is the main part of the reconstruction algorithm. To speed up the computation of Fourier transforms for the 3D reconstruction, ODTbrain employs the FFTW library [13].

In practice, for a successfull reconstruction a pre-processing step is required: the presented implementation requires a sinogram whose projections are distributed equally from 0 to 360° and a rotational axis that is located at the center of each projection. Phase retrieval and image alignment are not part of the reconstruction process and therefore not discussed in this paper.

## Results and discussion

### Finite-difference time-domain simulations

In order to test the ODTbrain library, we created tomographic data sets from artificial cell phantoms. We performed 2D and 3D finite-difference time-domain (FDTD) simulations with the software MEEP [14] to obtain *in silico* sinograms containing phase and amplitude of the scattered wave field. We decided to use the FDTD technique, because it describes the vectorial propagation of light according to the Maxwell equations, allowing us to test the approximations against the most realistic *in silico* data sets for optical tomography. The MEEP implementation of FDTD is also very robust, easy to use, and thus more practical than simulations based on the exact generalized Lorenz-Mie theory [15, 16]. In order to obtain an image as it would be seen through a microscope, we numerically autofocus the measured field from one wavelength (1 *λ*) behind the phantom to the center of the phantom by minimizing the gradient of the field amplitude [17–19]. Note that the resulting field at the detector is effectively measured with a numerical aperture of *N* *A*=1. Thus, the FDTD simulation technique is ideal for the investigation of arbitrary cell phantom geometries.

**Fig. 1:**
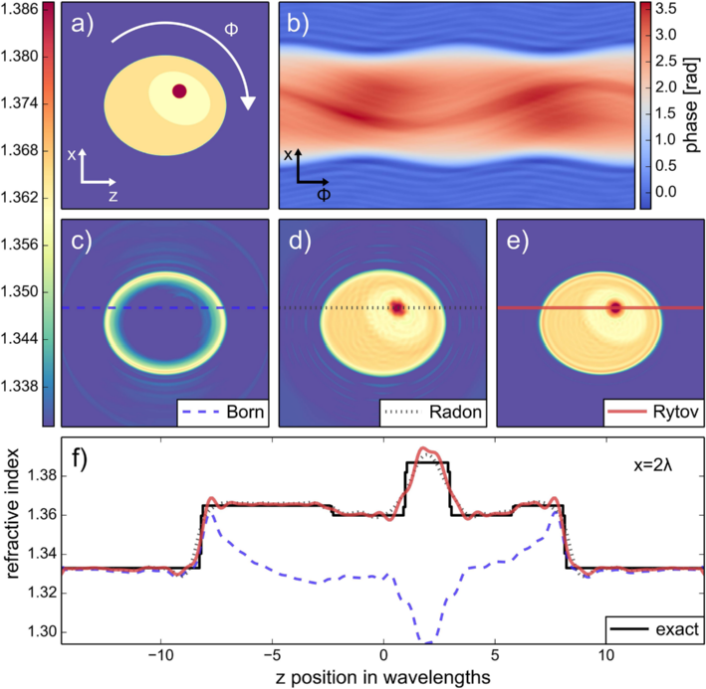
Refractive index reconstruction from 2D FDTD simulations. **a** False color image of the cell phantom with refractive index values of the medium (blue, 1.333), the cytoplasm (orange, 1.365), the nucleus (yellow, 1.360), and the nucleolus (red, 1.387). **b** As the cell phantom is rotated through the angle *ϕ*, from 0 to 360 degrees, the projections of a plane wave (sinogram) are computed with FDTD simulations. Displayed is the phase of the background-corrected and numerically refocused field that is used for the reconstruction. Red values indicate high phase retardation and blue values indicate low phase retardation. **c**, **d**, **e** Reconstruction of the cell phantom with the Born (**c**), Radon (**d**), and Rytov (**e**) approximations. **f** Line plots through the reconstructed cell phantom along the lines indicated in (**c**), (**d**), and (**e**). A total of 200 projections were used for the reconstruction

An exemplary 2D FDTD simulation is shown in Fig. 1. The figure depicts the 2D cell phantom (Fig. 1 a) and the phase of the sinogram which was computed using FDTD simulations (Fig. 1 b). The values of the refractive index for cytoplasm, nucleus, and nucleolus were taken from [20]. A detailed description of the 2D cell phantom is given in Additional file 1. The 2D reconstructions shown in Fig. 1 c, d, e and the corresponding line plots in Fig. 1 f illustrate the superiority of the Rytov approximation over the Born and Radon approximations. The Born approximation entirely fails to reproduce absolute refractive index values [7, 10, 11] and the Radon approximation produces smeared-out refractive index distributions, which becomes visible when comparing the shape of the nucleolus (red) in Fig. 1 d and e with a. The reconstruction with the Rytov and Born approximations were performed with ODTbrain, while the reonstruction with the Radon approximation was performed with the backprojection algorithm as implemented in [21]. The Rytov approximation yields the best reproduction of the initial phantom.

**Fig. 2:**
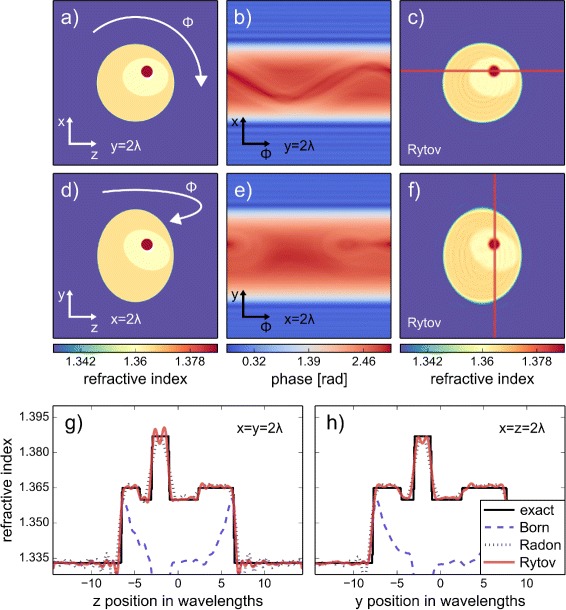
Refractive index reconstruction from 3D FDTD simulations. **a**, **d** Cross-sectional slices of the 3D cell phantom through the nucleolus located at *x*=*y*=*z*=2 *λ* from the center of the volume. The color scale is identical to that used in Fig. 1. **b**, **e** Sinogram slices matching the cross-sectional position of (**a**) and (**d**) for rotational position of *ϕ* ranging from 0 to 360 degrees; computed with FDTD simulations. **c**, **f** Cross-sectional slices of the reconstruction with the Rytov approximation at the same coordinates as in (**a**) and (**d**). **g**, **h** Line plots through the reconstructed cell phantom with the Born, Radon, and Rytov approximations. The positions of the line plots are shown in (**c**) and (**f**). A total of 200 projections were used for the reconstruction

The 3D analog to Fig. 1 is shown in Fig. 2. The cell phantom is rotated about the *y*-axis and the resulting 3D sinogram consists of 2D projections for the different rotational positions *ϕ* of the cell phantom. The line plots through the nucleolus of the reconstructed volume in Fig. 2 g and h show the same trend as the 2D line plots in Fig. 1 f. A visualization of the 3D cell phantom is given in the Additional file 2. The reconstructed slice shown in Fig. 2 f exhibits a directional blur that is visible at the upper and lower perimeter of the cell. This directional blur is the result of an incomplete data coverage in the object spectrum commonly referred to as missing apple-core artifacts [22]. Furthermore, it is known that the values at the center of the reconstruction volume along the axis of rotation are error-prone due to coherent noise in the measured sinogram [17]. We documented the artifacts resulting from the incomplete object spectrum and from the noise introduced during simulation and reconstruction in Additional file 3. As in the 2D case, the Rytov approximation yields the best reconstruction in the 3D case.

### Reconstruction quality measures

In order to obtain a measure for the reconstruction quality, we introduce two metrics; the normalized root-mean-square (RMS) error and the normalized total-variation (TV) error. We compute the normalized RMS error E_RMS_ according to

(1) $$ \mathrm{E}_{\text{RMS}} = \sqrt{ \frac{ \sum_{\text{vol}} \left(n_{\text{ph}} - n_{\text{rec}}\right)^{2} }{ \sum_{\text{vol}} \left(n_{\text{ph}} - 1 \right)^{2} } }   $$

where *n*_rec_ is the real-valued refractive index distribution of the reconstruction and *n*_ph_ that of the phantom. The summation $\sum _{\text {vol}}$ runs over all points that are within the circular (2D) or spherical (3D) reconstruction volume. Points that are outside of this reconstruction volume do not have contributions from all projections and are thus not considered. The RMS error quantifies the error in the refractive index at each point of the reconstruction.

As briefly mentioned in the previous section, the reconstruction with the Radon approximation leads to a blurred reconstruction. Furthermore, the Born approximation cannot reconstruct the correct magnitude of the refractive index values, but produces less blurry images than the Radon approximation. We quantify this blurriness by introducing the TV norm of the differences between phantom *n*_ph_ and reconstruction *n*_rec_. We compute the normalized TV error E_TV_ according to

(2) $$ \mathrm{E}_{\text{TV}} = \sqrt{ \frac{ \sum_{\text{vol}} \left(\text{TV}_{\text{avg}}^{N\mathrm{D}}\!\left(n_{\text{ph}} - n_{\text{rec}} \right) \right) }{ \sum_{\text{vol}} \left(n_{\text{ph}}-1\right)^{2} } }   $$

with an averaged TV norm computed for *N* = 2 or *N* = 3 dimensions according to the dimensionality of the simulation

(3) $$\begin{array}{@{}rcl@{}} \text{TV}_{\text{avg}}^{\mathrm{2D}}\!\left(n \right) &=& \frac{1}{2} \big[\mathrm{|grad}_{x}(n)| + |\text{grad}_{z}(n)| \big] \end{array} $$

(4) $$\begin{array}{@{}rcl@{}} \text{TV}_{\text{avg}}^{\mathrm{3D}}\!\left(n \right) &=& \frac{1}{3} \big[ |\text{grad}_{x}(n)| + |\text{grad}_{y}(n)| \,+\, |\text{grad}_{z}(n)| \big]. \end{array} $$

The normalization $\sum _{\text {vol}} \left (n_{\text {ph}}-1\right)^{2}$ implies a normalized RMS error of 100 %, when the reconstruction *n*_rec_ deviates from the phantom *n*_ph_ at magnitudes that are comparable to the difference between the phantom *n*_ph_ and a refractive index value of 1. In the same manner, the normalized TV error becomes 100 % if the averaged TV norm is equal to the squared difference between phantom *n*_ph_ and 1. The chosen RMS and TV metrics lead to error values that allow a direct comparison between the 2D and 3D reconstruction algorithms (see below).

### Quality dependence on total number of projections

**Fig. 3:**
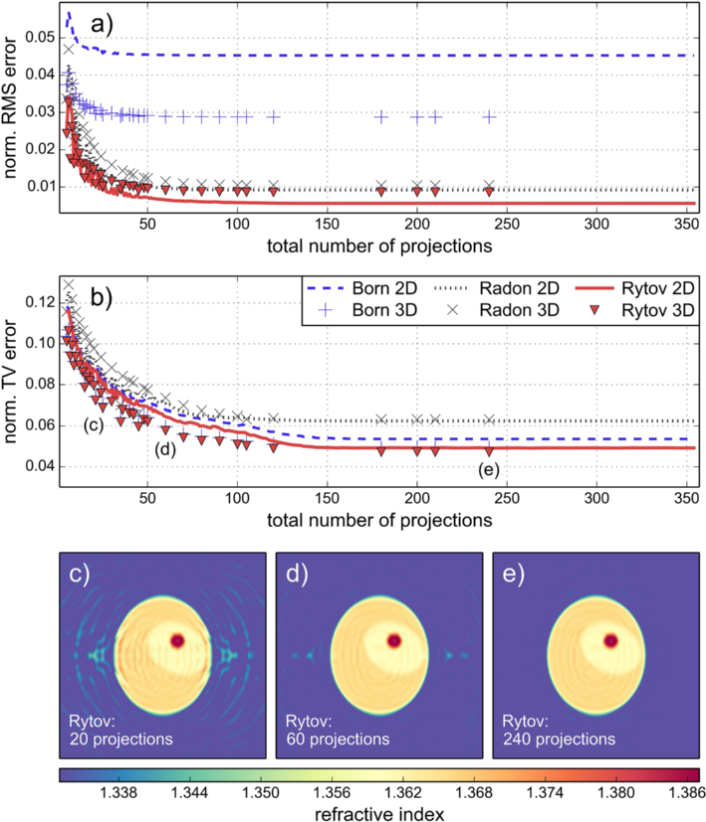
Dependence of reconstruction quality on the total number of projections. **a** Normalized root mean square (RMS) error in dependence of the total number of projections used for the reconstruction. The 2D (lines) and 3D (marker symbols) reconstructions were performed with the Born (blue), Radon (black), and Rytov (red) approximations. **b** Normalized total variation (TV) error in dependence of the total number of projections. **c**, **d**, **e** Slices through the 3D reconstruction volume for 20 (**c**), 60 (**d**), and 240 (**e**) projections. The corresponding slice for 200 total projections is shown in Fig. 2f. The diameter (2*a*=17 *λ*) and the refractive index values of the cell phantom are unchanged for all simulations

In order to determine the number of projections that are necessary for full-view and dense diffraction tomography, we performed 2D and 3D simulations with varying total numbers of projections, equally distributed between 0 and 360°. The cell phantoms used for the 2D and 3D simulations are those described above. The RMS and TV error in dependence of the total number of projections are shown in Fig. 3 a and b. At about 160 projections, both RMS and TV errors reach a plateau, which suggests that above this point, the reconstruction cannot be improved any further. For this reason, we set the number of projections to 200 for all subsequent simulations.

Besides the determination of the required number of projections, one can observe two other important facts in Fig. 3. First, the Rytov approximation results in the best reconstruction in 2D as well as in 3D. Second, the RMS and TV errors for the 3D reconstruction follow the RMS and TV errors of the 2D reconstruction. Apparently, the choice of the two error metrics defined in Eqs. 1 (RMS) and 2 (TV) results in similar error estimates for 2D and 3D reconstructions, independent of the number of projections. This similarity of the 2D and 3D errors suggests that the error of a 3D reconstruction may be inferred from the error of a 2D reconstruction. Even though no proof exists for such a connection, this observation potentially saves time or, in the case of limited computational resources, allows to make error estimates in 3D possible at all (see below).

### Quality dependence on refractive index variation

**Fig. 4:**
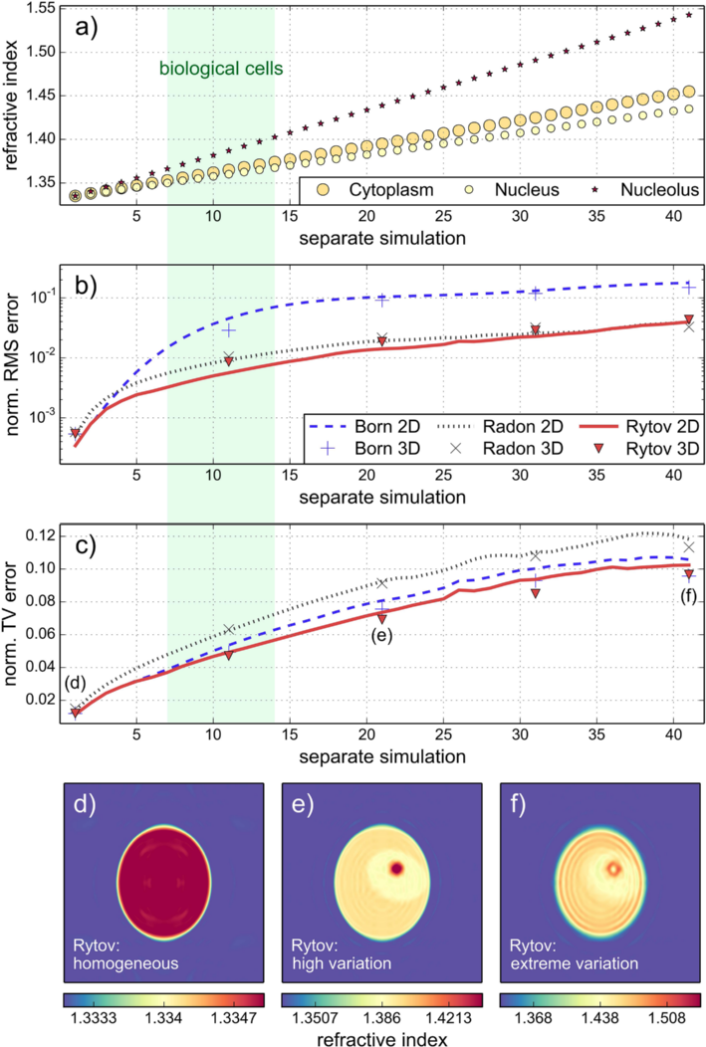
Dependence of reconstruction quality on the refractive index variation. **a** The refractive index values of the cell phantom range from 1.334 to 1.455 (cytoplasm), 1.435 (nucleus), and 1.543 (nucleolus) in a linear fashion. The approximate range of the refractive index for biological cells is marked in green. The refractive index of the medium is 1.333. **b** Normalized root mean square (RMS) error in dependence of the refractive index values shown in (**a**). **c** Normalized total variation (TV) error in dependence of the refractive index values shown in (**a**). **d**, **e**, **f** Cross sections of the 3D refractive index reconstruction with the Rytov approximation. The three simulations are labeled in (**c**). The diameter (2*a*=17 *λ*) of the cell phantom and the total number of projections (200) are unchanged for all simulations

In all the cases discussed above, we used fixed refractive index values for the cell phantom. Here, we show how the reconstruction quality varies with different refractive index values for cytoplasm, nucleus and nucleolus. Figure 4 a shows the different combinations of intracellular refractive index values (see figure caption). In the first simulation, the refractive index values of cytoplasm, nucleus, and nucleolus were set to a fixed value of just above water. The resulting RMS and TV errors are very small (Fig. 4 b and c, first simulation) which results in a good reconstruction of the homogeneous refractive index distribution (Fig. 4 d). The RMS and TV errors both increase as the refractive index values of the cell phantom increase. At refractive index values that are above the values for biological cells (1.36 – 1.39 [20]), the reconstruction of the cell phantom with the Rytov approximation is still successful (Fig. 4 e). At very large refractive index values (cytoplasm 1.455, nucleus 1.435, nucleolus 1.543), the reconstruction starts to show artifacts, as shown in Fig. 4 f. Quantitative line plots through the nucleolus are shown in Additional file 4. In comparison to Fig. 3, we again observe that the Rytov approximation yields the lowest reconstruction error and that the 3D error follows the 2D error. Furthermore, the Rytov approximation is valid across a large range of refractive index values, even outside of the regime of biological cells.

### Quality dependence on object size

**Fig. 5:**
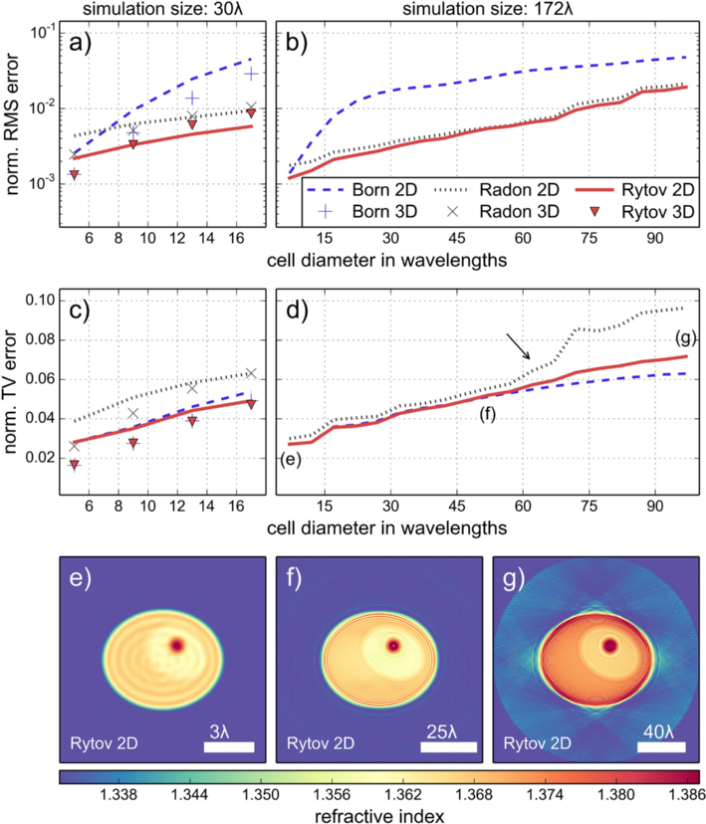
Dependence of reconstruction quality on the size of the cell. **a** Normalized root mean square (RMS) error in dependence of the cell diameter for two- and 3D reconstructions with the Born, Radon, and Rytov approximations. The extent of the simulation volume is 30 *λ*. **b** Same as in (**a**), except that the extent of the simulation volume is 172 *λ*. Due to computational limitations, a corresponding 3D simulation was not feasible with the FDTD method. **c** Normalized total variation (TV) error in dependence of the cell diameter corresponding to the values in (**a**). **d** Normalized TV error for the 2D series as described in (**b**). At the position indicated by the arrow we observed a sudden break down of the one-dimensional phase unwrapping algorithm. **e**, **f**, **g** 2D refractive index reconstructions with the Rytov approximation. The cell diameters are 7 *λ* (**e**), 52 *λ* (**f**), and 97 *λ* (**g**). The refractive index values of the cell phantom and the total number of projections (200) are unchanged for all simulations

The size of the cell phantom influences the reconstruction quality. For fixed resolution and detector size, small cells are more reliably reconstructed than large cells, because less pixel or voxel values have to be reconstructed. To illustrate this fact, we conducted simulations for different diameters of the cell phantom. In order to be able to compare the different reconstructions in terms of RMS and TV errors, the size of the simulation volume was kept constant. Due to limited computational power, a 3D simulation for cell diameters above 20 wavelengths was not feasible. Figure 5 shows the dependence of the reconstruction quality on the size of the cell. As noted in the previous sections, the RMS and TV errors of the 3D reconstruction follow that of the 2D reconstruction for the number of projections (Fig. 3) and for different refractive index distributions (Fig. 4). We make the same observation for different cell sizes (Fig. 5 a, c) when the simulation volume is small (30 *λ*). Therefore, we assume that the 2D errors depicted in Fig. 5 b and d may serve as an approximation for the 3D errors that we would observe for very large cells or small cell clusters. The graphs show that up until a cell diameter of 60 wavelengths the reconstruction with the Rytov approximation is very accurate (see also Additional file 5). Above this critical size, phase unwrapping errors lead to reconstruction artifacts. In summary, the correct reconstruction below object sizes of 60 wavelengths justifies the application of the Rytov approximation to 3D arrangements like biological cell clusters.

## Conclusions

The presented algorithm is an extension to optical projection tomography that takes into account diffraction of light due to the refractive index of the sample. We verified previous reports of the superiority of the Rytov approximation over the Born and Radon approximations in 2D for the 3D case. As predicted by the theory, the Born approximation breaks down quickly for large cells and for large RI changes [6]. We have shown that full-view, dense data sets must contain at least 160 projections to achieve the best possible reconstruction of a biological cell with a diameter of 17 wavelengths or less. In contrast, few-view and sparse data sets usually require some form of regularization (see [23, 24]). To improve reconstruction quality, the presented implementation in ODTbrain can be extended by regularization methods such as missing-angle artifact minimization, artifact removal by total variation minimization, and iterative reconstruction algorithms [24, 25].

The refractive index range covered successfully by the Rytov approximation is large and includes the range of values found in most biological cells (up to 1.40). Furthermore, the Rytov approximation yields good results for large objects (diameter ≈50 *λ*). This versatile validity makes the Rytov approximation interesting for the investigation of complex biological samples like cell clusters or even small embryos but also non-biological structures such as optical fibers.

## Availability and requirements

**Project name:** ODTbrain**Project home page:** http://odtbrain.craban.de **Operating system:** Platform independent**Programming language:** Python**Other requirements:** Python 2.7 or Python 3.4**License:** BSD (3 clause)**Any restrictions to use by non-academics:** None

## Additional files

Additional file 1
**Description of cell phantom for 2D FDTD simulations.** The schematic drawing shows the 2D refractive index phantom for the FDTD simulations. The cytoplasm with a refractive index of 1.365 (orange) is centered in the simulation volume (*P*_1_=(0,0)) and has major and minor axis of *a*_1_=8.5 *λ* and *b*_1_=7.0 *λ*. The nucleus with a refractive index of 1.360 is positioned off-center at *P*_2_=(2 *λ*,1 *λ*) with *a*_2_=4.5 *λ* and *b*_2_=3.5 *λ*. The nucleus is rotated with respect to the coordinate system at a fixed angle of *θ*=0.5 rad. The nucleolus with a refractive index of 1.387 is positioned at *P*_3_=(2 *λ*,2 *λ*) with a radius of *r*_3_=1 *λ*. (PNG 774 kb)

Additional file 2
**Description of cell phantom for 3D FDTD simulations.** The video is a visualization of the 3D refractive index phantom for the FDTD simulations. The values for the refractive indices, sizes, and positions for the 3D phantom are identical to that of the 2D phantom. The cytoplasm and nucleus of the 3D version of the phantom are prolate ellipsoids. (AVI 5140 kb)

Additional file 3
**Central cross-sections of 3D reconstruction with the Rytov approximation.** This figure supplements Fig. 2 with cross-sections and line plots through the center of the 3D volume. Along the axis of rotation (highlighted by two white lines), the reconstruction exhibits strong variations. (PNG 1003 kb)

Additional file 4
**Line plots of the 3D reconstruction for different magnitudes of the refractive index variation.** The figure is a quantitative representation of the three distributions of refractive index highlighted in (Fig. 4 d, e, f) with line plots through the nucleolus parallel to the minor (a, c, e) and the major axis (b, d, f). The reconstruction with the Born, Radon, and Rytov approximations are plotted. (PNG 1280 kb)

Additional file 5
**Line plots of the 2D reconstruction for different cell sizes.** The figure is a quantitative representation of the three distributions of refractive index highlighted in (Fig. 5 e, f, g) with line plots through the nucleolus as indicated in (Fig. 1 c, d, e). The reconstruction with the Born, Radon, and Rytov approximations are plotted. (PNG 1065 kb)

## Abbreviations

2D: Two-dimensional
3D: Three-dimensional
DHM: Digital holographic microscopy (quantitative phase imaging technique)
FDTD: Finite-difference time-domain (simulation technique used for forward process)
ODT: Optical diffraction tomography (e.g. backpropagation)
OPT: Optical projection tomography (e.g. backprojection)
RMS: Root-mean-square (used for error estimation)
TV: Total variation (used for error estimation)
