# Supplementary figures and images for: ODTbrain: a Python library for full-view, dense diffraction tomography
Source: BMC Bioinformatics. 2015 Nov 4;16:367. doi: 10.1186/s12859-015-0764-0 (PMC4634917; doi:10.1186/s12859-015-0764-0)

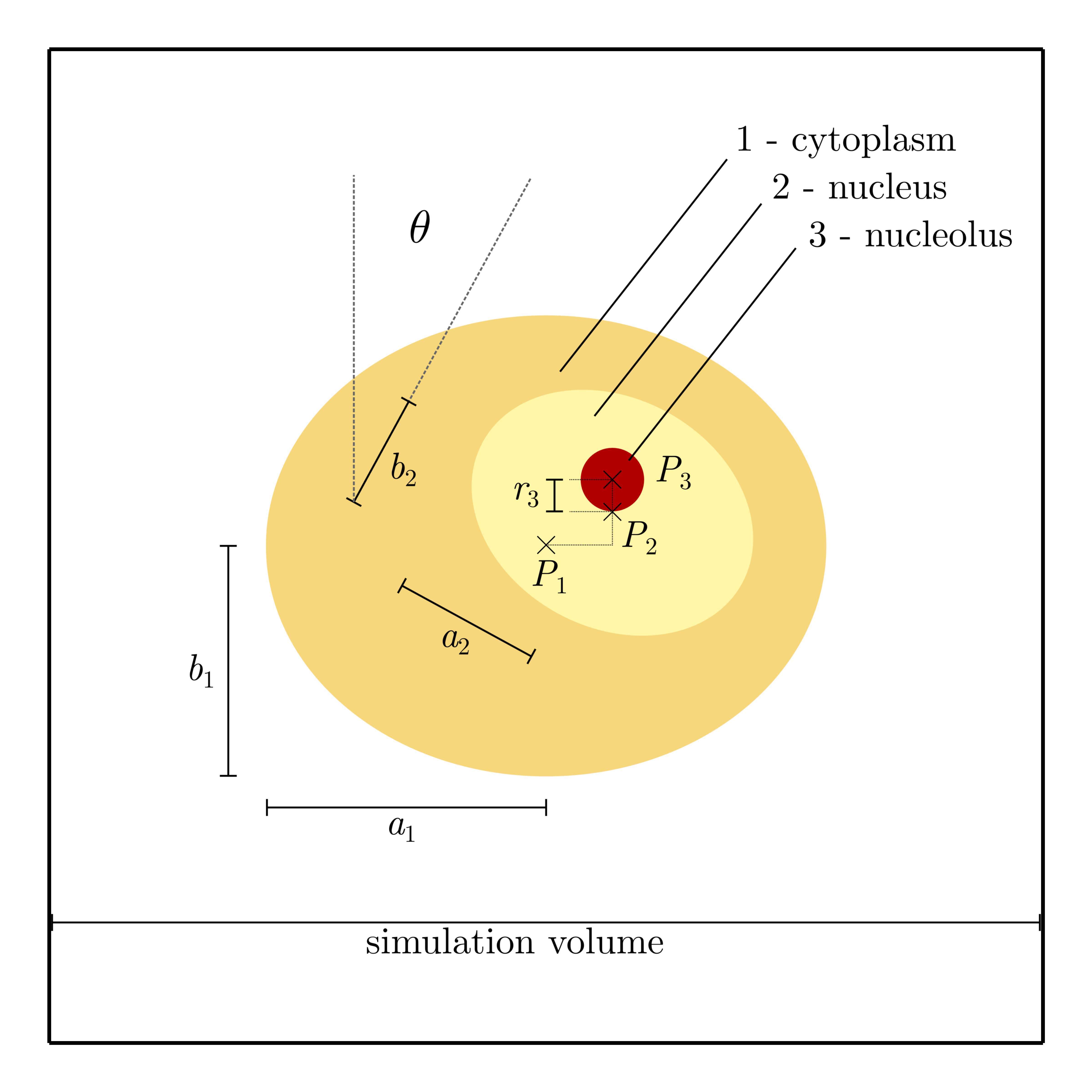

Supplement: Additional file 1 — Description of cell phantom for 2D FDTD simulations. The schematic drawing shows the 2D refractive index phantom for the FDTD simulations. The cytoplasm with a refractive index of 1.365 (orange) is centered in the simulation volume (P 1=(0,0)) and has major and minor axis of a 1=8.5 λ and b 1=7.0 λ. The nucleus with a refractive index of 1.360 is positioned off-center at P 2=(2 λ,1 λ) with a 2=4.5 λ and b 2=3.5 λ. The nucleus is rotated with respect to the coordinate system at a fixed angle of θ=0.5 rad. The nucleolus with a refractive index of 1.387 is positioned at P 3=(2 λ,2 λ) with a radius of r 3=1 λ. (PNG 774 kb) [file 12859_2015_764_MOESM1_ESM.png]

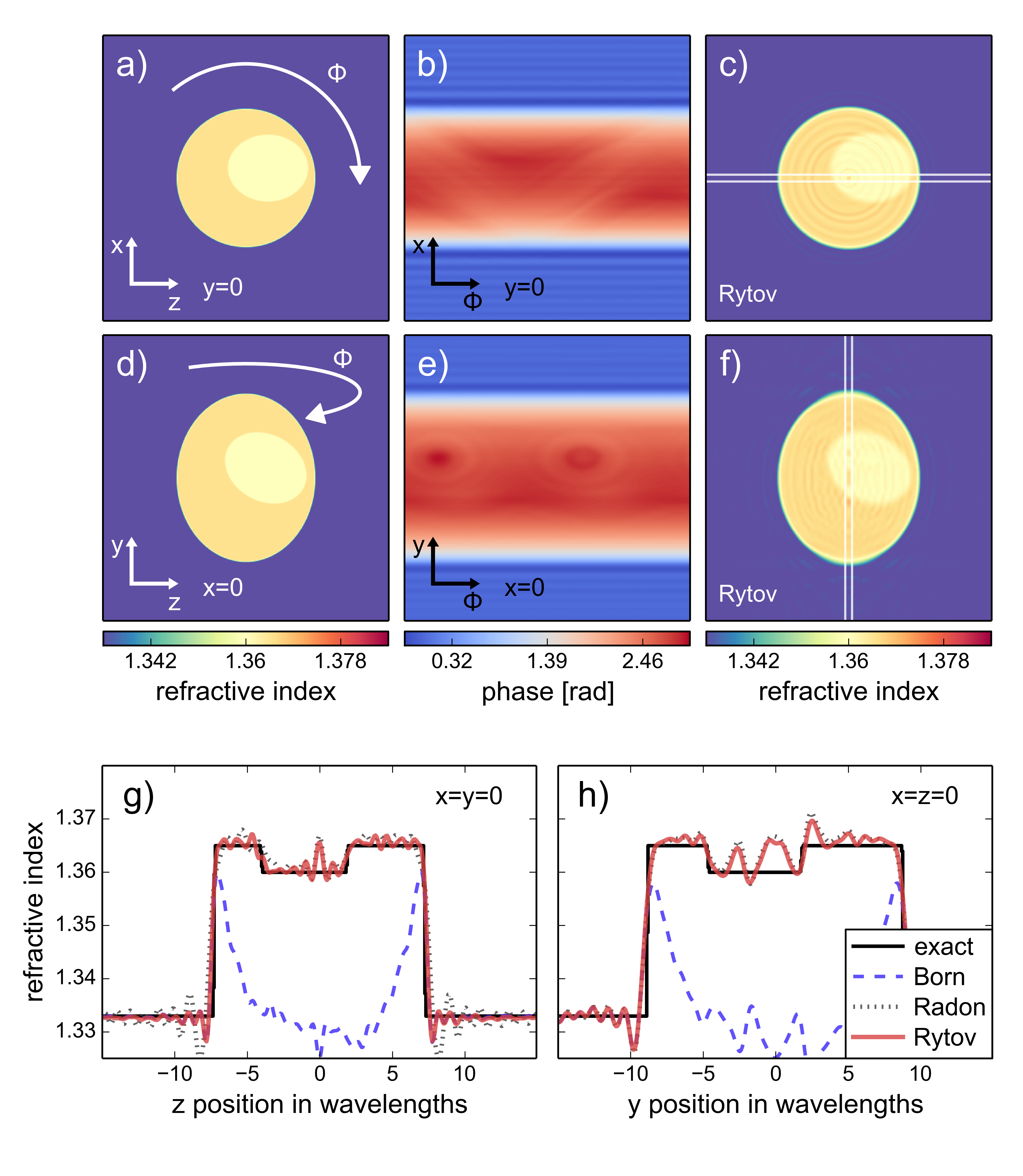

Supplement: Additional file 3 — Central cross-sections of 3D reconstruction with the Rytov approximation. This figure supplements Fig. 2 with cross-sections and line plots through the center of the 3D volume. Along the axis of rotation (highlighted by two white lines), the reconstruction exhibits strong variations. (PNG 1003 kb) [file 12859_2015_764_MOESM3_ESM.png]

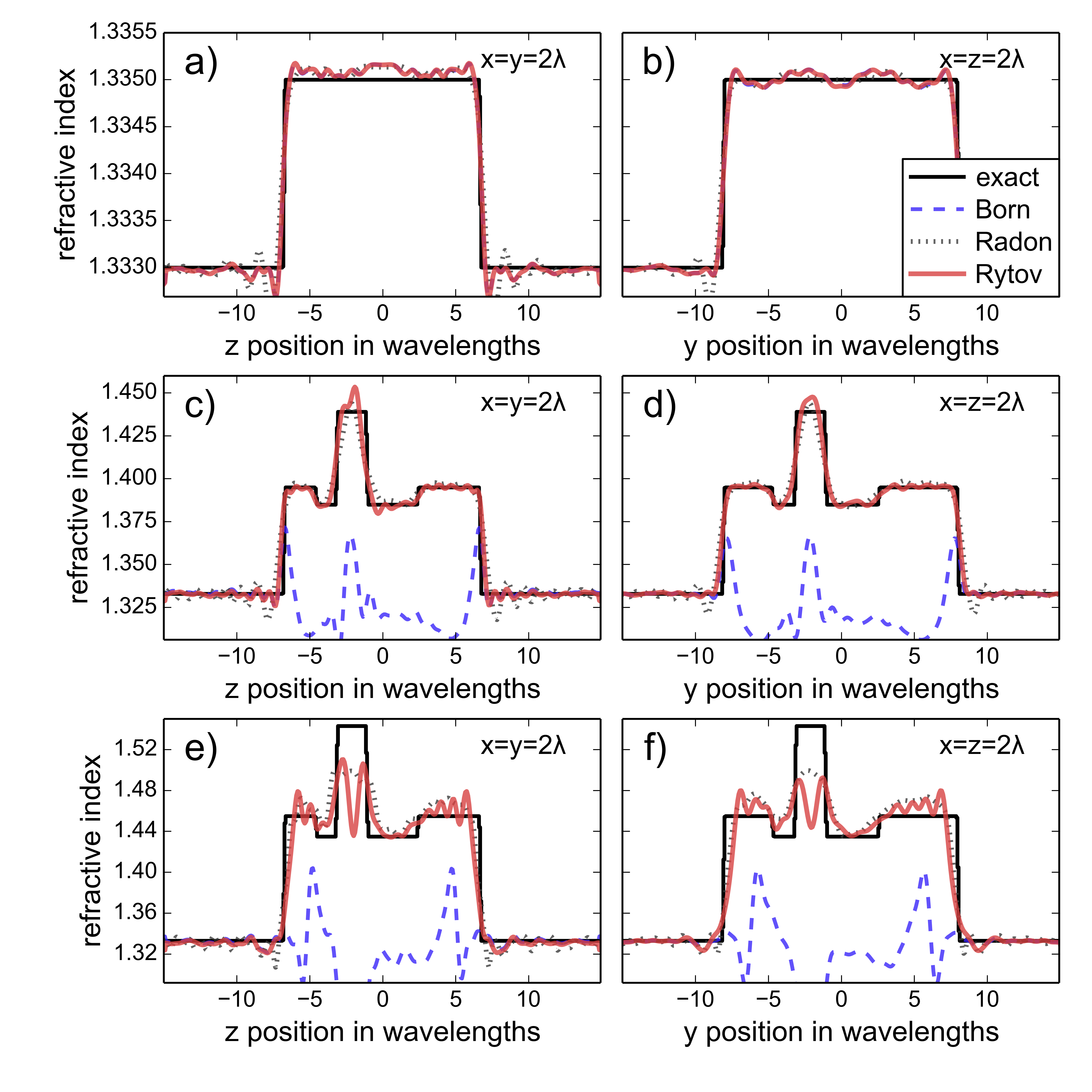

Supplement: Additional file 4 — Line plots of the 3D reconstruction for different magnitudes of the refractive index variation. The figure is a quantitative representation of the three distributions of refractive index highlighted in (Fig. 4 d, e, f) with line plots through the nucleolus parallel to the minor (a, c, e) and the major axis (b, d, f). The reconstruction with the Born, Radon, and Rytov approximations are plotted. (PNG 1280 kb) [file 12859_2015_764_MOESM4_ESM.png]

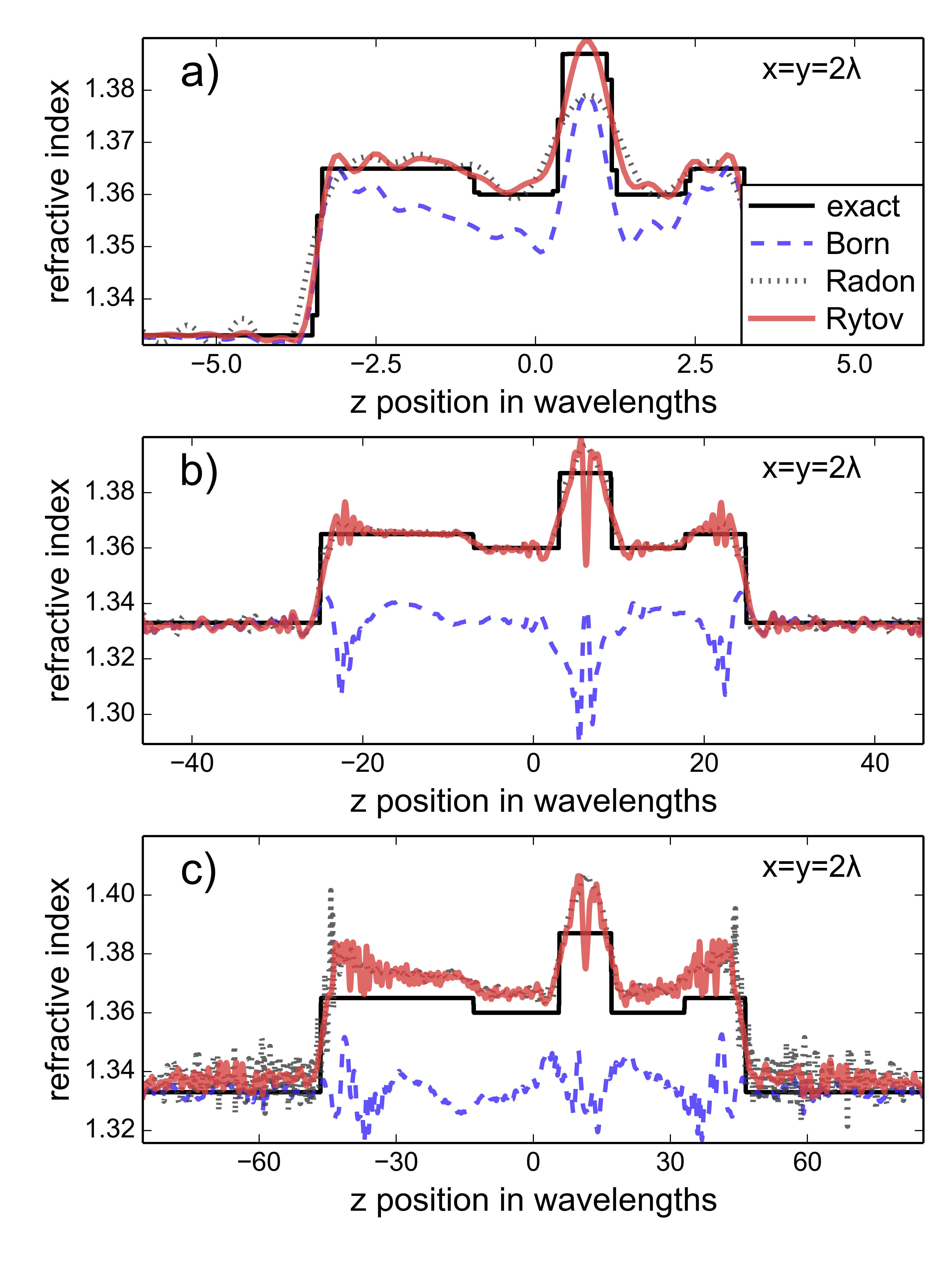

Supplement: Additional file 5 — Line plots of the 2D reconstruction for different cell sizes. The figure is a quantitative representation of the three distributions of refractive index highlighted in (Fig. 5 e, f, g) with line plots through the nucleolus as indicated in (Fig. 1 c, d, e). The reconstruction with the Born, Radon, and Rytov approximations are plotted. (PNG 1065 kb) [file 12859_2015_764_MOESM5_ESM.png]
